# Lifeday coverage of oral anticoagulants and one-year relative survival in patients with atrial fibrillation: a population-based study in Estonia

**DOI:** 10.1186/s12872-023-03415-4

**Published:** 2023-08-11

**Authors:** Priit Pauklin, Toomas Marandi, Mart Kals, Tiia Ainla, Katrin Martinson, Jaan Eha, Priit Kampus

**Affiliations:** 1 Department of Cardiology, Institute of Clinical Medicine, University of Tartu, 8 Puusepa Street, 50406 Tartu, Estonia; 2 Heart Clinic, Tartu University Hospital, 8 Puusepa Street, 50406 Tartu, Estonia; 3 Centre of Cardiology, North Estonia Medical Centre, 19 Sütiste Street, 13419 Tallinn, Estonia; 4 Estonian Genome Center, Institute of Genomics, University of Tartu, 23b Riia Street, 51010 Tartu, Estonia; 5 Linnamõisa Family Medicine Center, 16 Koskla Street, 10615 Tallinn, Estonia

**Keywords:** Atrial fibrillation, Anticoagulants, Stroke risk, Adherence to guidelines, Lifeday coverage

## Abstract

**Background:**

Routine oral anticoagulation (OAC) is recommended for almost all high-risk patients with atrial fibrillation, yet registries show that OACs are still underused. Our aim was to study the lifeday coverage (LDC) of OAC prescriptions and its relationship with one-year mortality rates of AF patients aged ≥ 65 in Estonia for the years 2019 and 2020.

**Methods:**

Medical data for AF patients aged ≥ 65 years from 2018 and alive as of 01.01.2019 (cohort I) and new AF documentation from 2019 and alive as of 01.01.2020 (cohort II) was obtained from the Health Insurance Fund’s electronic database. The data was linked to the nationwide Estonian Medical Prescription Centre’s database of prescribed OACs. For LDC analysis, daily doses of guideline-recommended OACs were used. The patients were categorized into three LDC groups: 0%, 1–79%, and ≥ 80%. The data was linked to the Estonian Causes of Death Registry to establish the date of death and mortality rate for the whole Estonian population aged ≥ 65.

**Results:**

There were 34,018 patients in cohort I and 9,175 patients with new AF documentation (cohort II), previously not included in cohort I. Of the patients, 77.7% and 68.6% had at least one prescription of OAC in cohorts I and II respectively. 57.4% in cohort I and 44.5% in cohort II had an LDC of ≥ 80%. The relative survival estimates at 1 year for LDC lifeday coverage groups 0%, 1–79%, and ≥ 80% were 91.2%, 98.2%, and 98.5% (cohort I), and 91.9%, 95.2%, and 97.6% (cohort II), respectively.

**Conclusions:**

Despite clear indications for OAC use, LDC is still insufficient and anticoagulation is underused for stroke prevention in Estonia. Further education of the medical community and patients is needed to achieve higher lifeday coverage of prescribed OACs.

## Background

Atrial fibrillation (AF) is the most common sustained cardiac arrhythmia in clinical practice and its prevalence increases with age [1]. AF is associated with substantial morbidity and mortality [1]. Routine use of oral anticoagulants (OACs) is recommended for patients with AF with CHA2DS2-VASc score values ≥ 2 (men) and ≥ 3 (women) for stroke prevention [1, 2].

In the last decade, vitamin K antagonists (VKAs) have been mostly replaced by non-vitamin K antagonist oral anticoagulants (NOACs) as the first-line management for stroke prevention [3]. Despite the ease of use and the wide availability of NOACs, registries of AF like the GARFIELD-AF [4, 5] show that OACs, in general, are still underused for stroke prevention [6]. In the GARFIELD-AF study, 38.0% of patients with an indication for OAC did not receive any anticoagulation [4]. The discontinuation rate was 13.0% in this registry, defined as the cessation of treatment for ≥ 7 days. Although 45.4% of patients restarted their therapy, they still had worse clinical outcomes with a higher chance of stroke or systemic embolism [7]. As most registries for studying AF patients rely on the self-reported use of OACs, their real-life use is not known [8]. In Estonia, a nationwide digital Medical Prescription Centre records the data of prescriptions and dispensed prescriptions for all prescription drugs in the country.

The main aim of this analysis was to study a novel and easily implementable way to characterize OAC prescriptions lifeday coverage (LDC) for stroke prevention for AF patients aged ≥ 65 years in Estonia and to investigate overall survival and AF-specific survival differences in LDC groups. Another objective was to characterize differences in concomitant diseases and the use of OACs between different LDC groups.

## Methods

### Data sources and study population

Estonia is a digitally advanced country with a population of 1.33 million [9]. Estonia also has a solidary health insurance system covering all permanent residents of Estonia or persons living with permits in Estonia who pay the social tax or are insured by the state [10]. All medical records and data about the prescription of drugs are digital and centralized in the Estonian Medical Prescription Centre’s database, which covers more than 99% of all prescriptions [11]. Every individual has a unique personal identification number. This allows us to link the diagnoses of each patient to information about the studied drugs. By adding information from the Estonian Causes of Death Registry for the date of death, it is possible to calculate the number of daily doses for each drug prescribed for every day alive, thereby providing information on LDC for each drug studied.

The study population consisted of patients aged ≥ 65 years with documented diagnoses of AF (I48, International Statistical Classification of Diseases 10th revision (ICD-10)) from the year 2018 and alive as of 01.01.2019 (cohort I) and patients with a new AF documentation from the year 2019 and alive as of 01.01.2020 (cohort II), previously not included in cohort I. The list of patients was obtained from the Estonian Health Insurance Fund’s (EHIF) database. This national database contains medical information about each inpatient and outpatient visit in Estonia, including the diagnoses according to ICD-10, and the coding for all medical services provided. The diagnoses of selected concomitant diseases (cancer (C00-C97), diabetes (E10, E11), hypertension (I10-I15), ischemic heart disease (I21, I22, I25.2), stroke (I63, I64, I69.3), peripheral artery disease (I70.2), renal insufficiency (N17-N19) and coronary stent (Z95.5)) were also obtained for the patients studied for the same period. The data from the nationwide Estonian Medical Prescription Centre about prescribed OACs for the period 01.01–31.12.2019 and 01.01–31.12.2020 or until death, if earlier, was also obtained for the same patients. For LDC analysis, daily doses of OACs recommended for stroke prevention were used as follows: warfarin (3 mg or 5 mg once a day (OD)) rivaroxaban (15 mg or 20 mg OD), apixaban 2.5 mg or 5 mg twice a day (BD)), dabigatran (110 mg or 150 mg BD) and edoxaban (30 mg or 60 mg OD). In case the dosing was different from the dosing recommended in guidelines [1], patients were categorized into the 0% group of OAC lifeday coverage. Because no information on the international normalized ratio (INR) was available for patients’ warfarin use, we considered dosing to be correct when at least 3 mg or 5 mg were used. Obtained data was linked to the Estonian Causes of Death Registry to establish the date of death. All-cause mortality rates by age and sex distribution were also obtained from the Estonian Causes of Death Registry to establish baseline mortality rate for the whole Estonian population aged ≥ 65 years for the years 2019 and 2020.

The study protocol was approved (document number 341/T-7) by the Research Ethics Committee of the University of Tartu. The study was conducted in accordance with the Declaration of Helsinki. In accordance with the Estonian Personal Data Protection Act and agreement from the Research Ethics Committee of the University of Tartu, individual informed consent to participate in this study was not needed, because patients were assigned a unique study number by the Health Insurance Fund and non-personalized data was released for analysis. All the data received from the Health Insurance Fund was anonymous and no individuals could be identified.

### Statistical analysis

Continuous variables are presented as means and standard deviation (SD) and categorical variables as frequencies and percentages.

LDC was defined as the proportion of days alive that are covered by daily OAC dose prescriptions recommended for stroke prevention. LDC was calculated for the one-year period considering also prescriptions from the previous year that overlapped with the study year. For patients who died during the one-year period, the LDC was calculated for the number of days alive. Due to lack of data, medication breaks were not taken into account in this study.

If a different OAC was prescribed for the patient during the study period, then the day of the new prescription was considered the switching day and the remaining doses of the previous OAC were excluded from LDC calculation.

The LDC groups of OACs were compared using Student’s t-test for continuous variables and the chi-squared test for categorical variables and the adjusted p-values were reported using the Bonferroni correction.

The all-cause mortality was assessed using the Kaplan-Meier method and differences between groups were tested by log-rank test. The expected survival was estimated using the age, sex and calendar year-matched general Estonian population data. Relative survival was calculated to estimate disease-specific survival as the ratio of the observed, all-cause survival of all the patients to the expected all-cause survival in the general population [12].

An underlying assumption of relative survival is that deaths associated with, or due to atrial fibrillation are an insignificant proportion of all deaths. Relative survival was estimated using the Pohar Perme non-parametric method, implemented in R package ‘relsurv’[13] and survival curves were compared using a log-rank type test [14]. All statistical analyses were performed using R [15], version 4.2.1.

## Results

In cohort I, there were 34,018 patients (60.3% females) with mean age 78.1 (SD = 7.3) years, and in cohort II, 9,175 patients (59.3% females) with mean age 77.5 (SD = 8.2) years. In cohorts I and II, 26,449 (77.7%) and 6,298 (68.6%) patients had at least one prescription of OAC, respectively. Patients in cohort II were younger (77.5 vs. 78.2 years, p < 0.001) and had a higher prevalence of concomitant diseases like cancer (14.8% vs. 13.9%, p = 0.024), renal insufficiency (11.5% s 9.7%, p < 0.001), coronary artery disease (13.4% vs. 12.3%, p = 0.003), and coronary stenting (8.2% vs. 7.0%, p < 0.001). Patients in cohort I had a higher prevalence of diabetes (23.4% vs. 22.3%, p = 0.021) and hypertension (88.4% vs. 86.7%, p < 0.001). The baseline characteristics of the study cohorts are presented in Table 1.

**Table 1:** Baseline characteristic of cohorts I and II.

Variable	Cohort I (2019)	Cohort II (2020)	p-value
Total patients, n	34,018	9175	
Mean age, years (SD)	78.1 (7.3)	77.5 (8.2)	< 0.001
Age 65–74, n (%) ≥75, n (%)	11,197 (32.9) 22,821 (67.1)	3551 (38.7) 5624 (61.3)	< 0.001
Female, n (%)	20,515 (60.3)	5438 (59.3)	0.074
Cancer, n (%)	4733 (13.9)	1362 (14.8)	0.024
Diabetes, n (%)	7968 (23.4)	2043 (22.3)	0.021
Hypertension, n (%)	30,059 (88.4)	7953 (86.7)	< 0.001
CAD, n (%)	4180 (12.3)	1234 (13.4)	0.003
Stroke, n (%)	3962 (11.6)	1136 (12.4)	0.055
PAD, n (%)	1764 (5.2)	501 (5.5)	0.307
Renal insufficiency, n (%)	3313 (9.7)	1051 (11.5)	< 0.001
Coronary stent, n %	2375 (7.0)	756 (8.2)	< 0.001
Use of ≥1 OACs, n (%)			
1	21,412 (76.2)	6327 (89.6)	< 0.001
2	6221 (22.1)	686 (9.7)	< 0.001
3	445 (1.6)	49 (0.7)	< 0.001
4	14 (0.0)	3 (0.0)	< 0.001
OAC monotherapy, n (%)			
-warfarin	2950 (13.8)	134 (2.1)	< 0.001
-dabigatran	2702 (12.6)	763 (12.1)	0.246
-rivaroxaban	8261 (38.6)	2311 (36.5)	0.003
-abixaban	7499 (35.0)	3092 (48.9)	< 0.001
-edoxaban	0 (0)	27 (0.4)	NA
OAC lifeday coverage			
0%	7569 (22.2)	2877 (31.4)	< 0.001
1–79%	6931 (20.4)	2215 (24.1)	
≥ 80%	19,518 (57.4)	4083 (44.5)	

OAC – oral anticoagulant

CAD – coronary artery disease

PAD – peripheral artery disease

**Fig. 1:**
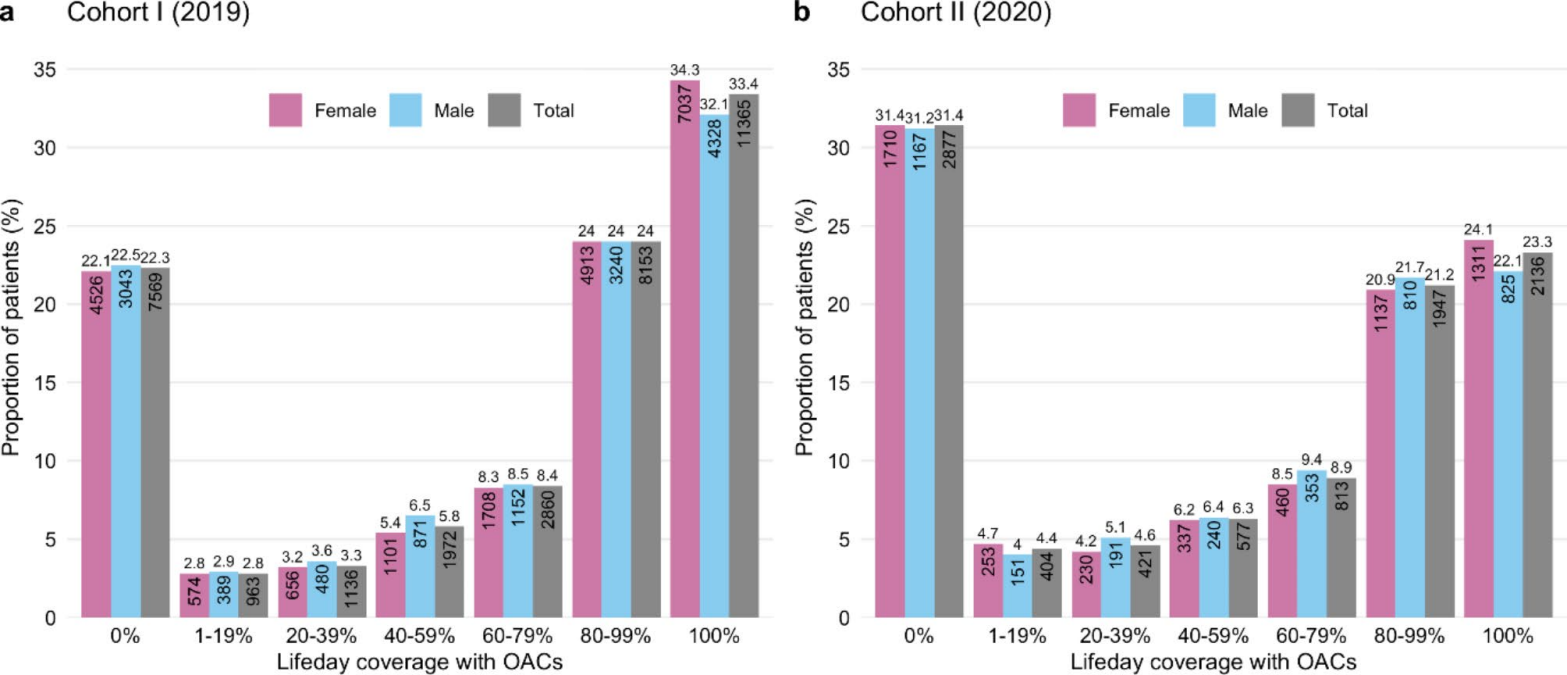
OAC prescriptions lifeday coverage Oral anticoagulant prescriptions lifeday coverage for cohorts I and II. The number of males, females, and total patients in different lifeday coverage groups is shown with the proportion of patients in percentages

The proportion of patients for whom the LDC of prescribed OACs was ≥ 80% was 57.4% in cohort I (55.6% of men and 57.7% of women) and 44.5% in cohort II (43.8% of men and 45.0% of women). The LDC distribution was U-shaped for both cohorts, where most of the patients were concentrated at the ends of the spectrum. The distribution of LDC is shown in Fig. 1.

We divided both cohorts into three groups by LDC proportions: 0%, 1–79%, and ≥ 80%. Patients in cohort I without any OAC prescriptions (0% group) showed a higher prevalence of stroke compared to the groups where OACs were prescribed. At the same time, a lower prevalence of diabetes and hypertension was noted in the LDC 0% group. This observation was not seen in cohort II.

There were 19,518 patients (57.4%) with a LDC of ≥ 80% in cohort I. A total of 14,448 patients (74%) had one prescribed OAC and 4,733 patients (24.2%) had two different prescribed OACs during the follow-up period of one year. For a small proportion of patients, three (1.7%) or four (0.1%) different OACs were prescribed during the study period in the LDC group ≥ 80%.

In cohort II, LDC for 4,083 patients (44.5%) was ≥ 80%. As in cohort I, most patients, 3,663 (89.7%) had one prescribed OAC, and 392 patients (9.5%) had two different prescribed OACs. Three or four different OACs were prescribed in 0.7% and 0.0% of the cases, respectively.

The characteristics of the different LDC groups regarding age, sex, concomitant disease, and different OAC use are presented in Tables 2 and 3.

**Table 2:** Comparison of different OAC lifeday coverage groups related to age, sex, and concomitant disease in cohort I

Variable	Cohort I (2019)			p-values for the differences between OAC lifeday coverage groups		
**OAC lifeday coverage**	0%	1–79%	≥ 80%	0% vs. 1–79%	0% vs. ≥ 80%	1–79% vs. ≥ 80%
**Patients, n (%)**	7569 (22.3)	6931 (20.3)	19,518 (57.4)			
**Mean age, y (SD)**	78.9 (7.9)	78.1 (7.1)	77.9 (7.0)	< 0.001	< 0.001	0.149
**Age 65–74, n (%)**	2384 (31.5)	2302 (33.2)	6511 (33.4)	0.086	0.011	1
**≥75, n (%)**	5185 (68.5)	4629 (66.8)	13,007 (66.6)			
**Female, n (%)**	4526 (59.8)	4039 (58.3)	11,950 (61.2)	0.195	0.095	< 0.001
**Cancer, n (%)**	1165 (15.4)	976 (14.1)	2592 (13.3)	0.084	< 0.001	0.292
**Diabetes, n (%)**	1560 (20.6)	1622 (23.4)	4786 (24.5)	< 0.001	< 0.001	0.192
**Hypertension, n (%)**	6423 (84.9)	6153 (88.8)	17,483 (89.6)	< 0.001	< 0.001	0.202
**CAD, n (%)**	959 (12.7)	913 (13.2)	2308 (11.8)	1	0.174	0.010
**Stroke, n (%)**	1025 (13.5)	743 (10.7)	2194 (11.2)	< 0.001	< 0.001	0.734
**PAD, n (%)**	431 (5.7)	374 (5.4)	959 (4.9)	1	0.029	0.366
**Renal insufficiency, n (%)**	818 (10.8)	697 (10.1)	1798 (9.2)	0.442	< 0.001	0.124
**Coronary stent, n (%)**	486 (6.4)	515 (7.4)	1374 (7.0)	0.054	0.225	0.870
**Use of ≥1 OACs, n (%)**						
- **1**	0 (0.0)	5420 (78.2)	14,448 (74.0)			< 0.001
**- 2**	0 (0.0)	1394 (20.1)	4733 (24.2)			< 0.001
**- 3**	0 (0.0)	115 (1.7)	325 (1.7)			1
**- 4**	0 (0.0)	2 (0.0)	12 (0.1)			NA
**OAC monotherapy, n (%)**						
**- warfarin**	0 (0.0)	514 (9.5)	2302 (15.9)			< 0.001
**- dabigatran**	0 (0.0)	728 (13.4)	1715 (11.9)			< 0.001
**- rivaroxaban**	0 (0.0)	1820 (33.6)	5863 (40.6)			< 0.001
**- abixaban**	0 (0.0)	2358 (43.5)	4568 (31.6)			< 0.001
**- edoxaban**	0 (0.0)	0 (0.0)	0 (0.0)			NA

OAC – oral anticoagulant

CAD – coronary artery disease

PAD – peripheral artery disease

SD – standard deviation

**Table 3:** Comparison of different OAC lifeday coverage groups related to age, sex, and concomitant disease in cohort II.

Variable	Cohort II (2020)			p-values for the differences between OAC lifeday coverage groups		
**OAC lifeday coverage**	0%	1–79%	≥ 80%	0% vs. 1–79%	0% vs. ≥ 80%	1–79% vs. ≥ 80%
**Patients, n (%)**	2877 (31.4)	2215 (24.1)	4083 (44.5)			
**Mean age, y (SD)**	78.4 (8.5)	77.2 (8.1)	77.0 (8.1)	< 0.001	< 0.001	0.610
**Age 65–74, n (%)**	1037 (36.0)	866 (39.1)	1648 (40.4)	0.083	0.001	1
**≥75, n (%)**	1840 (64.0)	1349 (60.9)	2435 (59.6)			
**Female, n (%)**	1710 (59.4)	1280 (57.8)	2448 (60.0)	0.743	1	0.300
**Cancer, n (%)**	451 (15.7)	311 (14.0)	600 (14.7)	0.341	0.825	1
**Diabetes, n (%)**	620 (21.6)	458 (20.7)	965 (23.6)	1	0.132	0.024
**Hypertension, n (%)**	2416 (84.0)	1919 (86.6)	3618 (88.6)	0.028	< 0.001	0.072
**CAD, n (%)**	396 (13.8)	277 (12.5)	561 (13.7)	0.609	1	0.542
**Stroke, n (%)**	352 (12.2)	217 (9.8)	567 (13.9)	0.021	0.147	< 0.001
**PAD, n (%)**	164 (5.7)	115 (5.2)	222 (5.4)	1	1	1
**Renal insufficiency, n (%)**	352 (12.2)	259 (11.7)	440 (10.8)	1	0.194	0.862
**Coronary stent, n (%)**	222 (7.7)	178 (8.0)	356 (8.7)	1	0.442	1
**Use of ≥ 1 OACs, n (%)**						
- **1**	0 (0.0)	1922 (86.8)	3663 (89.7)			< 0.001
**- 2**	0 (0.0)	272 (12.3)	392 (9.6)			0.001
**- 3**	0 (0.0)	19 (0.9)	27 (0.7)			0.472
**- 4**	0 (0.0)	2 (0.1)	1 (0.0)			NA
**OAC monotherapy, n (%)**						
**- warfarin**	0 (0.0)	33 (1.7)	92 (2.5)			0.048
**- dabigatran**	0 (0.0)	216 (11.2)	427 (11.7)			0.401
**- rivaroxaban**	0 (0.0)	629 (32.7)	1403 (38.3)			< 0.001
**- abixaban**	0 (0.0)	1019 (53.0)	1739 (47.5)			0.010
**- edoxaban**	0 (0.0)	25 (1.3)	2 (0.1)			NA

OAC – oral anticoagulant

CAD – coronary artery disease

PAD – peripheral artery disease

SD – standard deviation

**Fig. 2:**
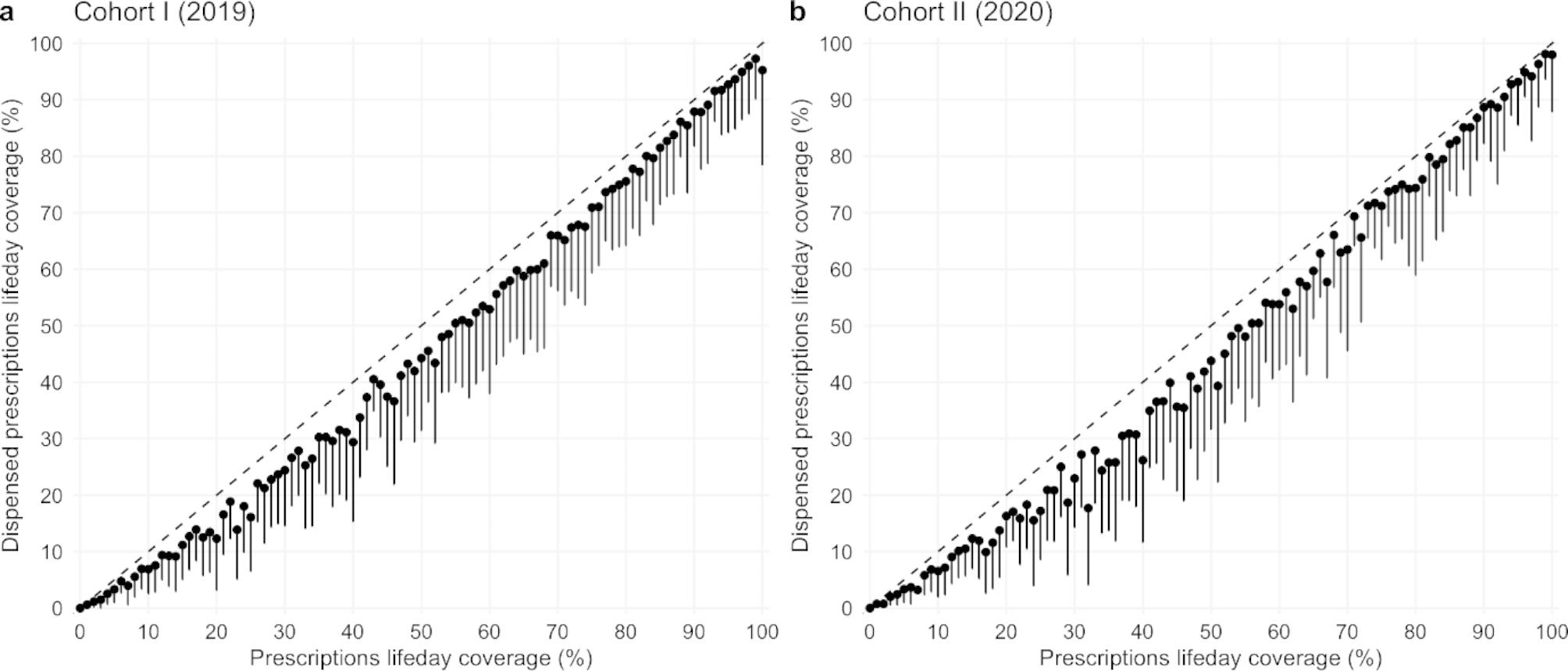
Differences between prescriptions and dispensed prescriptions OAC lifeday coverage Differences between prescribed and dispensed prescriptions OAC lifeday coverage for cohorts I and II. Patients are grouped by prescribed lifeday coverage in 1% increments. The diagonal line represents prescribed OAC lifeday coverage in percentages. Dots represent the proportion of dispensed prescriptions with a lower 95% confidence interval

While examining relationships between the OAC prescriptions LDC and the LDC of dispensed prescriptions in both cohorts, we found a clear correlation between them (Pearson’s correlation coefficients were 0.96 and 0.98 in cohorts I and II, respectively), indicating that most prescriptions were also dispensed (Fig. 2).

During the one-year follow-up period, 3,042 patients (9.0%; 1,279 men and 1,762 women) and 961 patients (10.5%; 411 men and 550 women) died in cohort I (2019) and cohort II (2020), respectively. The observed survival rates at 1 year for LDC groups 0%, 1–79% and ≥ 80% were 85.3% (95% CI 84.5–86.1%), 92.3% (95% CI 91.7–93.0%), and 92.6% (95% CI 92.5–93.2%) (cohort I) and 85.9% (95% CI 84.6–87.2%), 89.8% (95% CI 88.5–91.0%), and 92.0 (95% CI 91.2–92.9%) (cohort II), respectively. The all-cause mortality rate for the Estonian population aged ≥ 65 years was 4.7% for both years (Tables 4 and 5).

**Table 4:** The observed and relative survival estimates at 1 year for LDC groups in cohort I

	Patients	Observed mean survival % (95% CI)	p-values for observed survival curve differences*	Expected mean survival (%)	Relative survival % (95% CI)	p-value for net survival curve differences*
LDC groups						
**0%**	7569	85.3 (84.5–86.1)	-	95.3	91.2 (90.4–92.1)	-
**1–79%**	6931	92.3 (91.7–93.0)	< 0.001	95.3	98.2 (97.6–98.9)	< 0.001
**≥80%**	19,518	92.9 (92.5–93.2)	< 0.001	95.3	98.5 (98.1–98.9)	< 0.001
**Age groups**						
**65–74**	11,197	95.3 (94.9–95.7)	-	97.8	97.8 (97.4–98.2)	-
**≥75**	22,821	89.0 (88.6–89.4)	< 0.001	92.6	96.3 (95.9–96.8)	< 0.001
**Sex**						
**Female**	20,515	91.4 (91.0-91.8)	-	97.2	96.7 (96.3–97.2)	-
**Male**	13,503	90.5 (90.0–91.0)	0.006	98.1	96.9 (96.4–97.4)	0.629

* p-values are not adjusted for multiple testing

LDC – lifeday coverage

CI – confidence interval

**Table 5:** The observed and relative survival estimates at 1 year for LDC groups in cohort II.

	Patients	Observed mean survival % (95% CI)	p-values for observed survival curve differences*	Expected mean survival (%)	Relative survival % (95% CI)	p-values for net survival curve differences*
LDC groups						
**0%**	2877	85.9 (84.6–87.2)	-	95.3	91.9 (90.5–93.3)	-
**1–79%**	2215	89.8 (88.5–91.0)	< 0.001	95.3	95.2 (93.9–96.6)	< 0.001
**≥80%**	4083	92.0 (91.2–92.9)	< 0.001	95.3	97.6 (96.7–98.5)	< 0.001
**Age groups**						
**65–74**	3551	94.3 (93.6–95.1)	-	97.8	96.6 (95.8–97.4)	-
**≥75**	5624	86.5 (85.7–87.4)	< 0.001	92.4	94.3 (93.4–95.3)	< 0.001
**Sex**						
**Female**	5438	89.9 (89.1–90.7)	-	95.7	95.4 (94.5–96.1)	-
**Male**	3737	89.0 (88.0–90.0)	0.138	94.4	95.0 (93.9–96.1)	0.581

* p-values are not adjusted for multiple testing

LDC – lifeday coverage

CI – confidence interval

The relative survival estimates at 1 year for LDC groups 0%, 1–79%, and ≥ 80% were 91.2% (95% CI 90.4–92.1%), 98.2% (95% CI 97.6–98.9%), and 98.5% (95% CI 98.1–98.9%) (cohort I) and 91.9% (95% CI 90.5–93.3%), 95.2% (95% CI 93.9–96.6%), and 97.6% (95% CI 96.7–98.5%) (cohort II), respectively (Tables 4 and 5).

In both cohorts, observed and relative survival were significantly lower in LDC 0% groups compared to other LDC groups (all p-values < 0.001). The relative survival of male patients was similar to that of female patients, but younger patients (65–74 years) tend to have increased relative survival compared to older patients (≥ 75 years) (Tables 4 and 5).

**Fig. 3:**
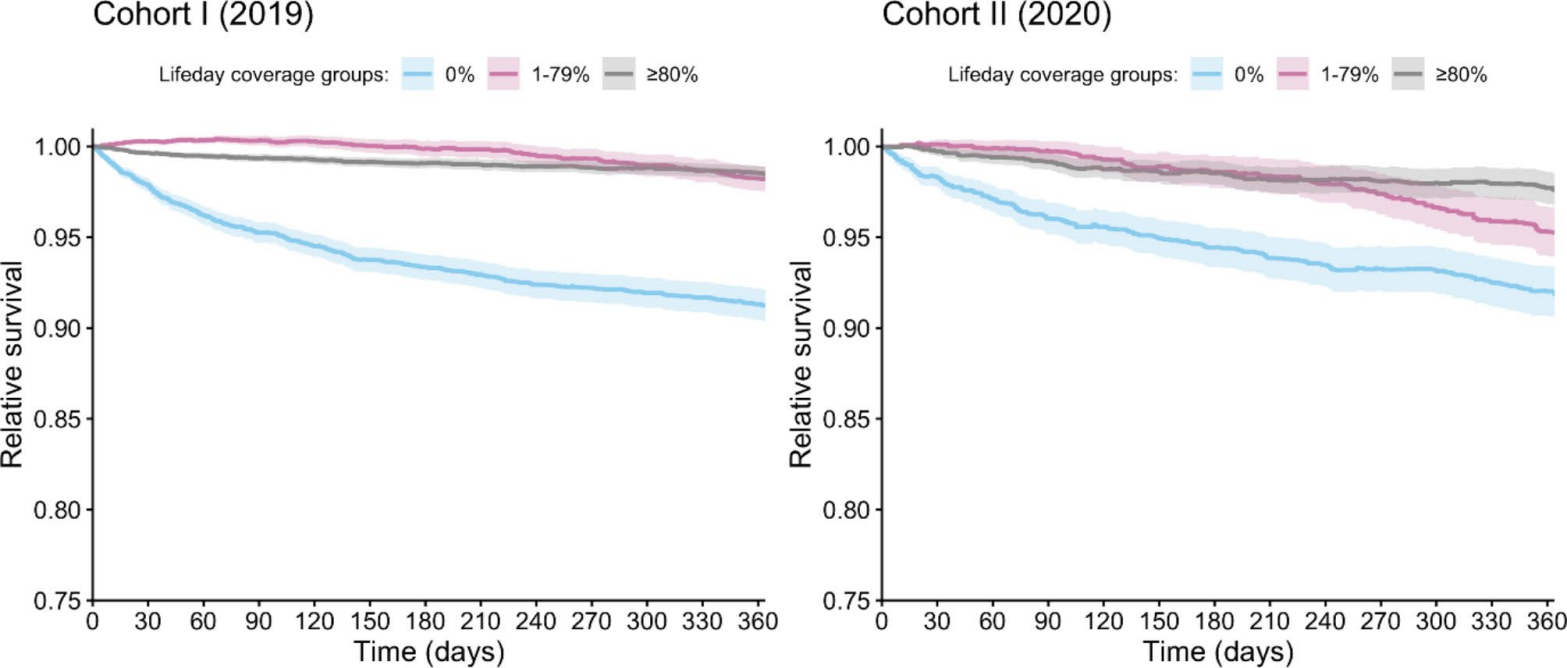
Comparison of relative survival curves by lifeday coverage groups among ≥ 65-year-old patients in Estonia Comparison of relative survival curves for ≥ 65-year-old patients in Estonia. Patients with one-year follow-ups were divided into three lifeday coverage groups. Relative survival was calculated as the ratio of the observed, all-cause survival of all the patients to the expected all-cause survival in the general population

Comparison of relative survival curves, defined as the ratio of the observed patient survival to the expected survival of a comparable group in the general population, matched to the patients with respect to age, sex, and calendar year, for the cohorts by the LDC groups for ≥ 65-year-old patients is shown in Fig. 3.

## Discussion

AF is strongly related to an increased risk of stroke [16–18] and anticoagulation is the cornerstone of stroke prevention for this population [19–22].

Our study describes an easily implementable method to characterize and assess the persistent use of OACs for the prevention of stroke. By using LDC analysis, we can obtain an accurate estimate of the real-world use of these drugs. The fact that the Estonian digital Medical Prescription Centre’s database contains the data of more than 99% of all prescriptions [11], means that the present study covers the whole Estonian population of 1.33 million [9]. To our knowledge, this type of prescription data analysis has not been performed in Estonia earlier.

As was shown, 77.7% and 68.6% of all AF patients aged ≥ 65 years had at least one OAC prescription in cohort I and cohort II, respectively. Data from the GARFIELD-AF and ORBIT-AF II registries have shown OAC use in 69% and 87% of the patients with CHA_2_DS_2_-VASc ≥ 2, respectively, which coincides roughly with our data about the overall use of OACs [4, 8].

However, these registries use self-reported data, which has many drawbacks [23]. The accuracy of data decreases with longer periods observed [24], and studies with other drugs have reported over- or underestimation of real-life use [25].

Other technologies like electronic tablet dispensers [26, 27] or QR-code based monitoring [28] have been developed to obtain a better estimate of tablet use, but these are mainly applicable in small-scale clinical trials and not suitable for monitoring the whole population [29].

Considering LDC, only 57.4% of the patients in cohort I and 44.5% of the patients in cohort II had OAC prescriptions, covering ≥ 80% of days alive. Data from Western European countries have shown that the persistence of NOAC therapy declined to 82% after one year. However, in persistent patients, 80% had a medication possession rate of ≥ 90% [30]. This result is in stark contrast with Estonian data, indicating the need to improve OAC use persistence.

As NOACs are fast-acting short-lasting drugs with a mean half-life ranging around 5–17 h [31], then missing of recommended doses or interruptions of continuous treatment can place the patients at higher stroke risk [32, 33]. This means that an optimum LDC of prescriptions should be aimed at reaching 100%. At the same time, studies with some NOACs have found that minimum effective coverage needs to be no less than 80% [32]. A 100% coverage was seen in only 33.4% and 23.3% of the patients from cohorts I and II, respectively. Further interventions and education of patients and healthcare providers are needed, to achieve higher coverage.

There was a statistical difference in mean age between the two cohorts due to the large sample size and a minor difference in mean and standard deviation, but this has no clinical implications. We saw more patients with concomitant renal insufficiency in cohort II (2020). A marked increase in the prescriptions of only one OAC during follow-up and a decrease in the use of 2 or 3 different OACs was noted in cohort II. As the prescription rate of warfarin was also reduced, then these changes seem to be due to the shifting from warfarin to NOACs, as well as to the wider availability of these drugs [34].

However, there occurred some differences between the LDC groups. Patients in the 0% groups were statistically older, but this difference was small. We did observe a higher prevalence of previously diagnosed stroke in the 0% group of cohort I. It could be hypothesized that the absence of OACs for patients with a previous stroke could be due to the fear of hemorrhagic events. However, at the same time, it is known that a previous stroke is an important risk factor for recurrent stroke [35], so careful assessment of OAC use or withholding the treatment is warranted [1]. This trend was not seen in cohort II.

We also saw a higher prevalence of cancer diagnosis among the population where no OACs were used in cohort I. As mentioned previously, we were not able to assess the prevalence of real contraindications for OAC use, so the reasons why the use of OACs is lower among cancer patients are not known. This might be associated with the use of low molecular weight heparin in this group, fear of bleeding, or frailty of the patients.

Surprisingly, we found a lower prevalence of hypertension in the 0% groups compared to the groups receiving OACs. A lower prevalence of diabetes was also seen in the 0% group in cohort I. This could be explained by the lower perceived risk of stroke among patients without concomitant diabetes or hypertension. At the same time, as the prevalence of hypertension (84.9% and 84.0%) and diabetes (20.6% and 21.6%) was still high in the 0% OAC groups of both cohorts, then the reasons why no anticoagulation was used for these patients is still uncertain.

In both cohorts, observed and relative survival were significantly lower in LDC 0% groups compared to other LDC groups indicating a beneficial effect of OAC use on survival in these patients [36].

Adherence to OAC therapy diminishes with time [37] and is related to increased stroke risk [33] One way to increase the LDC of OAC prescriptions could be on-demand or continuous assessment of the patients’ LDC by the family physician. Patients´ data from the Estonian Medical Prescription Centre’s database can be accessed by different medical software used in Estonia. An integrated tool that alarms the family physician or nurse of a lower-than-threshold LDC could be helpful to schedule a visit or remote consultation and renew the patient’s prescription. The same system could also be used for other medications that need to be taken regularly (e.g., anti-hypertensive or glycose-lowering drugs). Some small studies involving smartphone apps for prescription renewal reminders and educational materials have shown better adherence to OAC therapy in patients with AF [38, 39].

When examining the relationship between the data on prescriptions and dispensed prescriptions, we found that most prescribed drugs were also dispensed, meaning that an important culprit of low LDC seems to be the low rate of prescribing by the physician. Integrating individual patients’ LDC data in everyday clinical practice in an accessible and simple manner could improve long-term adherence to OAC therapy and other medications.

There are some limitations to this study that need to be addressed. As stated above, we could not assess the proportion of patients with true contraindications to OAC therapy. Therefore, we may have overestimated the proportion of patients in the 0% groups who should have received OAC therapy. Also, there might have been patients in the 0% category who were using low molecular weight heparin for stroke prevention that were not included in the study. Nor did we have access to INR monitoring data, so we were unable to assess the time in the therapeutic range for warfarin use for obtaining a reliable estimate of correct dosing.

There could be other confounding reasons for survival differences between the LDC groups that were not taken into account in this study as in-depth information was not available for every patient.

As our study focused on prescribed LDC, then the true estimate of the individual patients’ drug adherence remains out of the scope of the present study.

## Conclusions

Despite clear indications for OAC therapy, the LDC of prescriptions is still low and OACs are underused for stroke prevention in Estonia. Further education of the medical community and patients is needed to achieve higher coverage. Technical improvements and integrated tools that help to assess LDC in everyday clinical practice could improve patient care and adherence to chronic medical therapy.

## Data Availability

Due to the lack of a publicly accessible data repository, the datasets used and/or analyzed during the current study are available from the corresponding author upon reasonable request.

## Abbreviations

AF: Atrial fibrillation
CAD: Coronary artery disease
EHIF: Estonian Health Insurance Fund
ICD-10: International Statistical Classification of Diseases 10th version
INR: International normalized ratio
LDC: Lifeday coverage
NOAC: Non-vitamin K antagonist oral anticoagulant
OAC: Oral anticoagulant
PAD: Peripheral artery disease
SD: Standard deviation
VKA: Vitamin K antagonist
